# A case of psittacosis chlamydia pneumonia in patients with anti-infection drugs analysis and pharmaceutical care

**DOI:** 10.1097/MD.0000000000041187

**Published:** 2025-07-04

**Authors:** Hui He, Di Shen

**Affiliations:** a The People’s Hospital of Renshou County & The Second People's Hospital of Meishan City, Meishan, Sichuan Province, China; b Wenshan Prefecture Dermatology Prevention and Treatment Institute (Wenshan Prefecture Dermatology Specialist Hospital), Wenshan, Yunnan Province, China.

**Keywords:** anti-infective treatment, *Chlamydia psittaci*, clinical pharmacists, pharmaceutical care

## Abstract

**Rationale:**

Psittacosis pneumonia, caused by *Chlamydia psittaci*, is an underdiagnosed zoonotic infection. This case aims to demonstrate the efficacy of azithromycin as an alternative therapy and highlight the value of clinical pharmacists in managing drug-resistant infections.

**Patient concerns:**

A 56-year-old female poultry farmer presented with persistent high fever (38.5–39.0°C), chills, body aches, and fatigue for 2 weeks. Initial outpatient treatment with unspecified oral medication provided no relief.

**Diagnoses:**

Metagenomic next-generation sequencing of bronchoalveolar lavage fluid confirmed *C psittaci* and rhinovirus co-infection, explaining treatment failure with empirical piperacillin-tazobactam for presumed bacterial pneumonia.

**Interventions:**

After pathogen identification, intravenous azithromycin monotherapy (4.5 g total dose) was initiated. Clinical pharmacists collaborated on regimen design, monitored for QTc prolongation/electrolyte imbalances, and ensured safety during the 12-day course.

**Outcomes:**

Fever resolved within 48 hours; all symptoms improved by day 6. Follow-up computed tomography showed significant resolution of lung consolidation. The patient was discharged on day 11 with oral azithromycin continuation.

**Lessons:**

Azithromycin is effective as second-line therapy for severe psittacosis when tetracyclines are inaccessible. Metagenomic next-generation sequencing enables rapid diagnosis of atypical pathogens, while pharmacist-led pharmaceutical care optimizes antimicrobial selection and safety monitoring, particularly for cardiotoxic drugs.

## 1. Introduction

Psittacosis is a zoonotic infection caused by *Chlamydia psittaci* infection. Humans are infected with psittacosis mainly through contact with infected birds and their respiratory secretions or dust contaminated with feces.^[1]^ About 1% to 6.4% of community-acquired pneumonia is due to *C psittaci* infection.^[2]^ *C psittaci* pneumonia has a long incubation period and variable clinical manifestations, typical clinical manifestations are high fever, cold, cough, and pulmonary invasive lesions, and its clinical symptoms are similar to colds or respiratory infections. Secondly, *C psittaci* pneumonia lacks specific detection means, so it is easy to be ignored by clinicians and missed diagnosis or misdiagnosis.^[3,4]^ In recent years, with the widespread clinical application of metagenomics next-generation sequencing (mNGS), reports of *C psittaci* infection have also increased. We now report a case of *C psittaci* pneumonia confirmed by mNGS in our hospital as follows. Through retrospective analysis of clinical pharmacists’ participation in the whole process of diagnosis and treatment of a case of *C psittaci* pneumonia, this paper aims to provide ideas for the early diagnosis and treatment of the disease, and also provide references for clinical pharmacists’ pharmaceutical care, so as to ensure the safety of patients’ medication.

## 2. Case description

The patient, female, 56 years old, came to our hospital for treatment due to fever, chills, temperature measuring 38.5℃, accompanied by body aches, fatigue and other symptoms. Chief complaint: Two weeks before admission, fever, chills, and body temperature were measured at 38.5℃ without obvious causes, accompanied by body aches and fatigue, and the symptoms did not relieve by buying medicine orally at the local clinic (specific details are unknown). She was hospitalized in our hospital on June 6, 2023.

Physical examination on admission showed: body temperature 38.5℃, pulse 123 times/min, respiratory rate 23 times/min, blood pressure 112/64 mm Hg, respiratory activity in both lungs was consistent, lung percussion was silent, auscultation was clear, dry and wet rales were not heard in both lower lungs, and there was no pleural friction sound. Blood routine examination showed: white blood cell count 6.41 × 10^9^/L, neutrophil percentage 76.4%, platelet count 126 × 10^9^/L, C-reactive protein 57.5 mg/L. Combined with other examinations such as CT, the diagnosis was considered as community-acquired pneumonia, non-severe.

After admission, the patient was given symptomatic treatment with anti-infection, cough, expectorant and other drugs. During the hospitalization, the patient still had repeated high fever. The maximum temperature was measured at 39.0℃, and the temperature dropped to normal after symptomatic treatment. The symptoms did not improve significantly after anti-infection treatment with piperacillin sodium and tazobactam sodium.

Admission diagnosis: community-acquired pneumonia was not severe.

After admission, the patient underwent ECG monitoring and received oxygen inhalation. The blood gas test revealed a pH of 7.46, PaCO2 of 33.9 mm Hg, PaO2 of 78.3 mm Hg, K + level of 3.88 mmol/L, Na + level of 139.4 mmol/L, and blood glucose level of 8.0 mmol/L. The initial treatment regimen included piperacillin and tazobactam for anti-infection therapy, acetaminophen for fever reduction, vitamin C injection, and a 5% glucose supplement.

On June 8th, 2023 (the 3rd day after admission), the patient experienced recurrent fever from the previous night to the following morning with a peak temperature reaching 38.3℃. After taking an oral suspension of D-ibuprofen (6 mL), the body temperature gradually returned to normalcy but was accompanied by symptoms such as dizziness, numbness, body aches, and fatigue; Additionally experiencing poor mental state, sleep quality, and appetite. The fever persisted without showing signs of abating. Relevant auxiliary examinations including bronchoscopy and alveolar lavage fluid sampling were completed for further evaluation.

On June 10th, 2023 (the 5th day after admission), the patient no longer exhibited fever, dizziness, numbness, body aches, or fatigue. Her mental status improved along with her diet. During medical history inquiry, the patient mentioned exposure to air conditioning prior to illness onset as well as prolonged poultry farming in the past.

On June 11th, 2023 (the 6th day of admission), the patient no longer exhibited symptoms such as fever, dizziness, numbness, body aches and fatigue. Her mental state and dietary intake were satisfactory. The NGS gene test results of bronchoalveolar lavage fluid indicated high confidence for *C psittaci* and rhinovirus type C. Considering the patient’s clinical presentation and previous exposure to chickens, the pathogen was suspected to be *C psittaci*. Therefore, azithromycin for injection was added to the anti-infective regimen.

On June 16th, 2023 (11 days after admission), the patient remained asymptomatic with regards to fever, chills, body aches or fatigue. The patient’s condition improved significantly leading to discharge on June 16th. Azithromycin was prescribed for an additional week as part of the anti-infective treatment plan while also emphasizing rest and enhancing immune function. Table 1 outlines antibiotic usage during hospitalization and post-discharge.

**Table 1:** Antimicrobial drug usage during hospitalization and upon discharge of patients.

Medication name	Dosage	Administration	Start date	End date
Injection of Piperacillin Sodium and Tazobactam Sodium	4.5 g	ivgtt, q8h	June 6th	June 16th
Azithromycin for Injection	0.5 g	ivgtt, qd	June 11th	June 16th
Azithromycin tablets	0.5 g	po, qd	June 17th	June 23th

**Figure 1.**
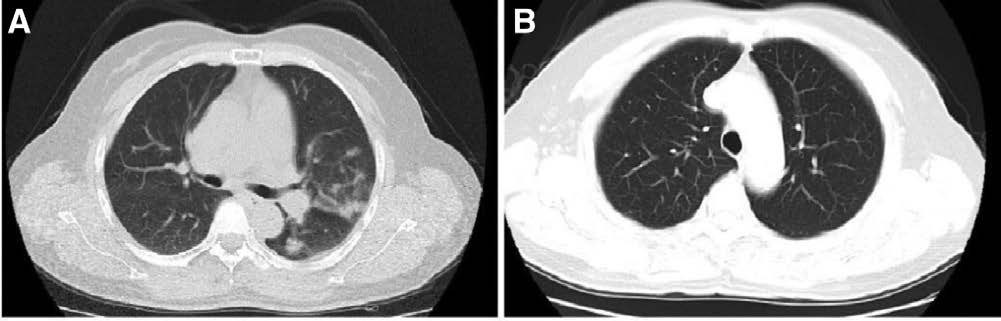
Chest CT of the patients during the treatment. (A) Outpatient checkups; (B) 14 days after azithromycin therapy.

The results of the CT examination on June 23, 2023, compared with the CT images from June 6, 2023, show that the lingular segment and lower lobe of the left lung were absorbed and lung consolidation was alleviated (Fig. 1A and B).

## 3. Discussion

### 3.1. Epidemiology of *C psittaci* pneumonia

*C psittaci*, an obligate intracellular gram-negative pathogen, is prevalent in avian species (particularly parrots and pigeons) as well as mammals. Aerosols generated from the feathers and feces of infected birds can be inhaled by humans, leading to respiratory infection. *C psittaci* pneumonia is recognized as a causative agent of community-acquired pneumonia.^[5]^ According to Hogerwerf et al’s study,^[1]^ *C psittaci* accounted for 1.03% of all cases of community-acquired pneumonia. Another study on patients with severe community-acquired pneumonia reported an incidence rate of *C psittaci* as high as 8%.^[6]^

The incubation period for *C psittaci* infection is protracted, typically ranging from 1 to 2 weeks.^[7]^ Human infection with *C psittaci* primarily presents as respiratory system diseases, although clinical manifestations can vary. *C psittaci* exhibits strong pathogenicity, often causing symptoms resembling influenza, such as chills, sore throat, and headache. Severe cases can result in complications including pneumonia, endocarditis, and encephalitis, posing a significant risk to life. In this study, the patient exhibited fever and chills as clinical manifestations but did not show improvement after self-administering amoxicillin. These symptoms represent the more prevalent clinical manifestations of infection with *C psittaci*.

### 3.2. Treatment of psittacosis chlamydophila pneumonia

According to recommended guidelines both domestically and internationally, the primary treatment for psittacosis chlamydophila pneumonia infection is tetracycline antibiotics (such as tetracycline, doxycycline, and minocycline). Due to its favorable in vivo activity and pharmacokinetic profile, doxycycline is widely utilized in clinical practice for treating psittacosis chlamydophila pneumonia. Reports have indicated that patients with psittacosis chlamydophila pneumonia experienced fever relief within 48 hours of commencing anti-infection treatment with doxycycline.^[8]^ Donati et al discovered that minocycline could inhibit 90% of Chlamydophila growth at a minimum drug concentration of 0.06 mg·L^−1^, which proved that minocycline also exhibits promising therapeutic effects on chlamydophila.^[9]^ In cases where tetracycline drugs are contraindicated, macrolides (such as erythromycin and azithromycin) can be used as second-line treatment drugs. Existing research have shown high resistance of chlamydophila to quinolone drugs; therefore, the recommendation for quinolones is not strong.^[10]^ In this particular study, the patient received piperacillin sodium and tazobactam treatment for 4 days while hospitalized; however, her condition continued to worsen.

After the patient was diagnosed with chlamydophila infection, intravenous azithromycin was administered for anti-infection therapy due to the unavailability of intravenous routes for doxycycline and tigecycline in the hospital setting.

Azithromycin, as a second-generation macrolide antibiotic, can inhibit bacterial protein synthesis and prevent the formation of 50S ribosomal subunits by binding to the sensitive bacterial ribosome 50S subunit. It highly inhibits bacterial growth and is used to treat various infections. Previous research have shown that macrolide antibiotics have high intracellular concentrations and are effective in treating *Chlamydophila pneumoniae* pneumonia.^[9]^ In this study, the patient was treated with azithromycin for 12 days, and the anti-infective regimen was adjusted promptly for concomitant infections. After anti-infective and symptomatic treatment, the patient’s symptoms such as chills and fever improved significantly, infection indicators returned to normal, and reexamination of lung CT showed significant absorption of pulmonary inflammation, confirming the effectiveness of the anti-infective regimen. This provides medication reference for clinical treatment of *C pneumoniae* pneumonia patients. The optimal treatment course for *C pneumoniae* pneumonia has not been determined yet. However, Guo Ying et al’s research suggests a recommended course duration of 10 to 21 days; whether prolonging anti-infective treatment is beneficial in preventing recurrence of *C pneumoniae* pneumonia has not been confirmed.^[7]^ The “Diagnosis and Treatment Guidelines for Community-Acquired Pneumonia (2016 Edition)” indicates that the usual course duration for mild to moderate CAP patients is 5 to 7 days; severe cases or those with extrapulmonary complications may require appropriately prolonged anti-infective therapy. For atypical pathogens with slow response to treatment, the course duration can be extended to 10 to 14 days; if infected with pathogenic bacteria such as *Staphylococcus aureus*, *Pseudomonas aeruginosa* or anaerobic bacteria which easily cause necrosis in lung tissue, the anti-infective course can be extended to 14 to 21 days.^[11]^ This indicates that the duration of anti-infective therapy for CAP should be based on disease severity, speed of relief, complications, and different pathogens involved; it should not solely rely on the degree of absorption of pulmonary shadows as an indication to discontinue antimicrobial drugs.

### 3.3. Medication monitoring

The patient was diagnosed with community-acquired pneumonia upon admission, which is non-severe. In order to ensure the safe use of medication for the patient, the clinical pharmacist should provide pharmaceutical care for the patient’s antibiotic therapy. Upon physical examination after admission, the absolute value of white blood cells was 5.38 × 109/L, with a neutrophil percentage of 76.8% and a lymphocyte percentage of 15.1%. The absolute value of lymphocytes was 0.81 × 109/L, and the high-sensitivity C-reactive protein level was 45.9 mg/L. On June 6th, 2023, following admission, the clinical pharmacist recommended Piperacillin/Tazobactam Sodium (4.5 mg). Piperacillin/Tazobactam Sodium is composed of Tazobactam as a beta-lactamase inhibitor and Piperacillin as a broad-spectrum penicillin antibiotic that achieves bactericidal effects by inhibiting bacterial cell wall synthesis; Tazobactam inhibits beta-lactamases to protect Piperacillin from enzymatic degradation, thereby enhancing its antibacterial activity. After consulting product information and literature data, it was found that injection of Piperacillin/Tazobactam Sodium may lead to gastrointestinal reactions such as diarrhea, nausea, and vomiting as well as adverse effects on the hematologic system (decreased white blood cell and platelet counts) and skin appendages (rash, itching). Therefore, during hospitalization post-administration monitoring focused on these potential adverse reactions. The patient did not experience any gastrointestinal adverse reactions during treatment nor did they exhibit damage to their skin or blood system indicators within normal range: white blood cell count at 5.38 × 10^9^/L and platelet count at 13.8 × 10^9^/L throughout their hospital stay. Upon discharge on June16th, 2023, the patient was advised to undergo regular follow-up blood tests after discharge while continuing medication in order to monitor for fever or abnormal hematologic responses.

Azithromycin is a second-generation macrolide antibiotic. Its antibacterial mechanism involves irreversibly binding to the target site of the 50S subunit of bacterial ribosomes, selectively inhibiting bacterial protein synthesis and achieving bacteriostatic effects. TISDALE reported that azithromycin may lead to cardiovascular adverse reactions such as prolonged corrected QT interval, severe arrhythmia, and cardiac-related death in clinical applications.^[12]^ Prolonged corrected QT interval can lead to torsades de pointes ventricular tachycardia, posing a threat to patient’s life. Studies have shown that azithromycin can cause corrected QT interval prolongation with an incidence rate of approximately 0.79%. Therefore, clinical pharmacists should monitor medication use to ensure patient safety.^[13]^ The patient in this study is a middle-aged woman with no history of cardiovascular disease and can use azithromycin under the supervision of a clinical pharmacist; it should be discontinued if there is prolonged corrected QT interval or other cardiac adverse events. At the same time, monitoring the patient’s water and electrolyte balance is necessary to maintain blood potassium, magnesium, calcium, phosphorus within normal ranges in order to reduce the likelihood of adverse events occurring. During hospitalization, the corrected QT interval values remained within normal range without prolongation or occurrence of cardiac adverse events.

Despite the insights provided in this case report on psittacosis chlamydia pneumonia in patients undergoing anti-infection drug therapy and pharmaceutical care, several limitations must be acknowledged. The single-case nature of the report restricts the generalizability of the findings to broader patient populations. Moreover, the case report relies heavily on the accuracy and completeness of medical records, which can be subject to errors or omissions. These limitations underscore the need for caution in interpreting the results and emphasize the importance of further research, including larger-scale studies with prospective designs and control groups, to confirm and expand upon the observations reported herein.

## 4. Conclusion

When humans contract Psittacosis chlamydia, it can develop into Psittacosis chlamydia pneumonia if misdiagnosed or underdiagnosed. With the extensive application of mNGS in clinical practice, the accuracy and timeliness of diagnosing *C psittaci* infection have been significantly enhanced. Clinicians and pharmacists can formulate individualized anti-infective treatment plans for patients based on the characteristics of the patients, medications, and pathogenic bacteria. Although tetracyclines have been widely regarded as the first choice for the treatment of *C psittaci* pneumonia, this study reveals that azithromycin also demonstrates remarkable efficacy as a second-line agent. This study not only provides new evidence for the selection of therapeutic drugs for *C psittaci* pneumonia but also offers new hope for those patients who are intolerant to tetracycline antibiotics or do not respond to the treatment. In the future, with the deepening of research, we anticipate providing a more solid theoretical basis for the selection of clinical drugs for the treatment of *C psittaci* pneumonia.

## Author contributions

**Conceptualization:** Di Shen.

**Data curation:** Di Shen.

**Methodology:** Hui He.

**Project administration:** Di Shen.

**Resources:** Hui He.

**Supervision:** Hui He.

**Visualization:** Hui He.

**Writing – original draft:** Hui He.

**Writing – review & editing:** Di Shen.
